# A Head of State Leading by Example

**DOI:** 10.3201/eid2810.AC2810

**Published:** 2022-10

**Authors:** Terence Chorba, José Esparza

**Affiliations:** Centers for Disease Control and Prevention, Atlanta, Georgia, USA (T. Chorba);; University of Maryland School of Medicine, Baltimore, Maryland, USA (J. Esparza)

**Keywords:** About the cover, art and medicine, numismatics, medals, immunization, variolation, smallpox, orthopoxvirus, viruses, public health, history, a head of state leading by example, Medal of Catherine II (1729‒1796), celebrating smallpox immunization efforts in Russia in 1768, Catherine the Great, Timothy Ivanov, Thomas Dimsdale, Russia

**Figure 1:**
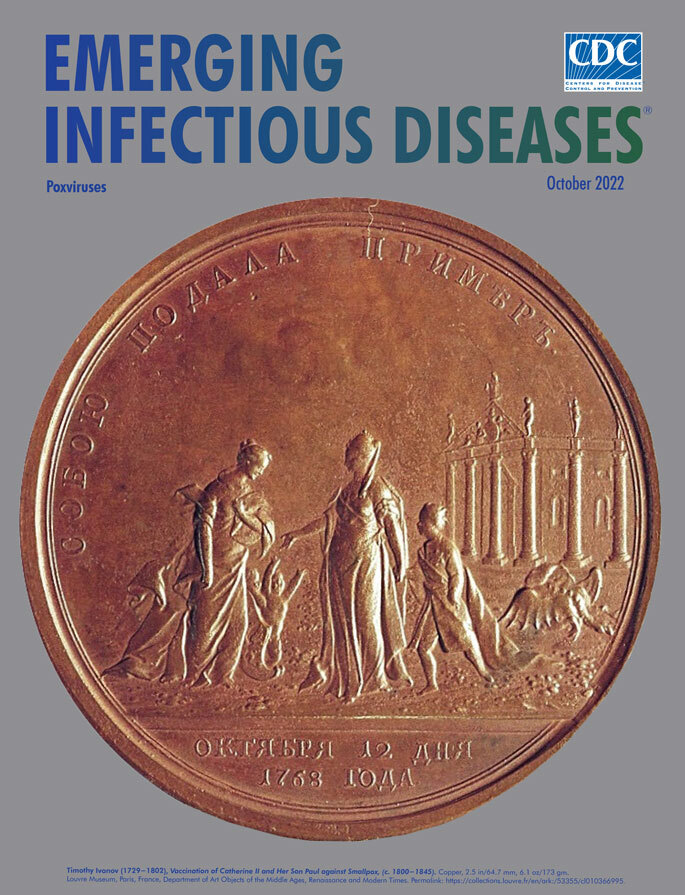
**Timothy Ivanov (1729−1802), *Inoculation of Catherine II and Her Son Paul against Smallpox*, (c. 1770−1800).** Copper, 2.5 in/64.7 mm, 6.1 oz/173 gm. Louvre Museum, Department of Art Objects of the Middle Ages, Renaissance and Modern Times. Permalink: https://collections.louvre.fr/en/ark:/53355/cl010366995

Before the introduction of smallpox vaccine, variolation was practiced as a preventive measure (i.e., deliberate infection with smallpox to provide immunity), most commonly by inserting or rubbing material from smallpox lesions into the skin of uninfected persons. Most persons thus infected would get a milder case of smallpox as the virus was generally introduced via the skin rather than via the respiratory route, as is the case of natural exposure. Infection occurring in this manner could still be transmitted by droplets to others who could develop a full-blown case of smallpox. Variolation developed over several centuries in many different sites including China, India, Sudan, Asia Minor, and Britain. Because variolation was reputed to have risk of inducing severe disease, variolation hesitancy existed long before the smallpox vaccine and its associated vaccine hesitancy. Inoculation with materials putatively derived from cowpox lesions (vaccination) or from horsepox lesions (equination) was a welcomed advance because it was safer and did not present the hazard of onward transmission of smallpox to the contacts of recipients. 

On the cover of this month’s journal is an image of a copper medal, executed by the Russian sculptor Timothy Ivanov (1729‒1802). Ivanov created several medals celebrating the contributions of Catherine II (1729–1796), also known as Catherine the Great, whose reign (1762‒1796) was that of Russia’s longest-ruling female leader. This medal was struck to honor those most distinguished in Russia’s mass immunization efforts against smallpox at the turn of the 18th into the 19th centuries. On the obverse (Figure), a crowned right-facing bust of Catherine is surrounded by a legend that reads *Б[ОЖИЕЮ]* *∙ М[ИЛОСТЬЮ]* *∙ ЕКАТЕРИНА II ИМПЕРАТ[РИЦА] ∙ И САМОДЕРЖ[ИЦА] ∙ ВСЕРОСС[ИЙСКАЯ]* (By the grace of God, Catherine II, Empress and absolute ruler of all Russia). Underneath the bust is inscribed *TИМOΘЕИ. ÏВАН[ОВЬ]* (Timothy Ivan[ov]). On the reverse (featured on the cover) is a female figure, presumably Catherine herself, holding the hand of her son, Paul Petrovich (later Tsar Paul I), encountering another female figure, perhaps Russia itself, and two small children. Representing disease, a dead hydra lies in the background below the colonnade and pediment of a classical-style building; these figures are surrounded by a legend: *COБOЮ ПOДАΛА ПPИМҌPЪ* (She herself set the example). Beneath is the Russian lettering for “October 12 in the year 1768,” the date on which Catherine was inoculated with smallpox material.

**Figure 2:**
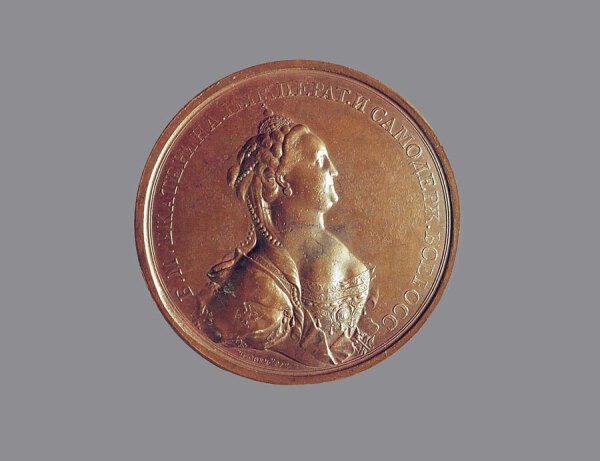
Obverse of medal featured on the cover art. Timothy Ivanov (1729−1802), *Bust of Catherine II*, (c. 1770−1800). Copper, 2.5 in/64.7 mm, 6.1 oz/173 gm. Louvre Museum, Department of Art Objects of the Middle Ages, Renaissance and Modern Times. Permalink: https://collections.louvre.fr/en/ark:/53355/cl010366995

Catherine’s efforts focused on the high endemicity of smallpox in Russia after the death of one of her ministers and after corresponding with Voltaire, another proponent of variolation. In 1768, Catherine recruited Thomas Dimsdale, an English physician who had published on inoculation methods and on variolation. On October 12, the empress was variolated in great secrecy. She then developed a mild case of smallpox, from which she recovered in 2 weeks. Her son, Paul, was variolated with material from one of her own smallpox pustules. However, as a precaution, in case her subjects were to hold Dimsdale accountable if she were to become gravely ill or die after variolation, Catherine had arranged for a yacht to be stationed temporarily in the Gulf of Finland to convey Dimsdale to a site out of danger, if needed. News of the success of Catherine’s procedure was then widely disseminated and Dimsdale inoculated more than 140 members of her court. The response to Catherine’s successful experiment was widespread uptake of the procedure elsewhere in Russia, and a burst of interest in funding hospitals and training programs and improving healthcare in general. 

The use of medals as a decorative form of jewelry dates to the 4th century bce, but from the Renaissance into the 19th century, the portrait medal flourished as a distinct art form to honor public figures. Medals have also been commonly used to commemorate public events or to reward worthy individuals. More than 20,000 medals are in the domain of medical numismatics, many of which commemorate discoveries in infectious diseases and the ends of epidemics. What sets Catherine’s medal apart is its uniqueness as a tool of public health advocacy. 

In 1796, English physician Edward Jenner inoculated a child with fluid extracted from human cowpox lesions. After the same child was later inoculated with material obtained from a human smallpox lesion, the child exhibited no clinical symptoms of smallpox, demonstrating that receipt of cowpox inoculation was protective against smallpox. The procedure was successful because cowpox virus and smallpox virus both belong to the same family (*Poxviridae*) and genus (*Orthopoxvirus*) of viruses; some *Orthopoxvirus* species generate cross-immunity in humans against subsequent infection with certain other *Orthopoxvirus* species. Another example of cross-immunity was observed after monkeypox, another *Orthopoxvirus* species, was identified in human populations. In the 1980s, when greater numbers of persons in Zaire (now the Democratic Republic of the Congo) had received smallpox vaccine, its prior receipt was found to confer considerable protection in close contacts of persons with monkeypox and in reducing the severity of disease associated with monkeypox. This phenomenon has been noted in considerations of vaccine development and risk in the current monkeypox outbreak.

The work of Dimsdale in pursuing variolation, the work of Catherine II in mobilizing the support of influential nobles to overcome inoculation hesitancy, and the work of Jenner in developing the first vaccine against smallpox all took place many decades before the germ theory of disease was conceived of and demonstrated by Edwin Klebs, Louis Pasteur, Robert Koch, and their disciples. As public recognition of the science and its success increased, variolation was replaced by the less risky practice of vaccination and inoculation hesitancy decreased with the concomitant decrease in associated adverse events. 
